# Construction of 3D-Printed Sodium Alginate/Chitosan/Halloysite Nanotube Composites as Adsorbents of Methylene Blue

**DOI:** 10.3390/molecules29071609

**Published:** 2024-04-03

**Authors:** Jinjie Luo, Anping Ji, Guofeng Xia, Lizi Liu, Juan Yan

**Affiliations:** Department of Mechanical Engineering, Chongqing Three Gorges University, Chongqing 404120, China; ji-anping@sanxiau.edu.cn (A.J.); xiaguofeng@sanxiau.edu.cn (G.X.); lzliu@alu.cqu.edu.cn (L.L.); 20140048@sanxiau.edu.cn (J.Y.)

**Keywords:** halloysite nanotube, sodium alginate, 3D printing

## Abstract

In this study, sodium alginate/chitosan/halloysite nanotube composites were prepared by three-dimensional printing and characterized in terms of morphology, viscosity, thermal properties, and methylene blue (MB) adsorption performance. The high specific surface area and extensively microporous structure of these composites allowed for effective MB removal from wastewater; specifically, a removal efficiency of 80% was obtained after a 60 min treatment at an adsorbent loading of 1 g L^−1^ and an MB concentration of 80 mg L^−1^, while the maximum MB adsorption capacity equaled 376.3 mg g^−1^. Adsorption kinetics and isotherms were well described by quasi-second-order and Langmuir models, respectively. The composites largely retained their adsorption performance after five adsorption–desorption cycles and were concluded to hold great promise for MB removal from wastewater.

## 1. Introduction

In view of its increasing severity, the problem of water pollution has drawn much attention worldwide [1]. In particular, organic dyes (which originate from textiles, leather, paper, printed matter, food packaging, etc.) and their decomposition products are highly toxic water pollutants that inflict irreversible damage to humans, animals, and marine organisms [2]. Moreover, some organic dyes are non-degradable and thus strongly impact the environment and human health [3]. Consequently, the elimination of dyes from water resources is a matter of high practical significance, as manifested by the development of numerous water treatment technologies, such as chemical oxidation, flocculation–coagulation, precipitation, photocatalytic degradation, membrane filtration, and adsorption. Among these techniques, adsorption offers the advantages of high removal efficiency, simplicity of adsorbent fabrication, and design/operation flexibility [4]. Many currently known adsorbents for dye removal comprise smart carbon-based nanomaterials such as carbon nanotubes [5] and graphene [6]. However, as these materials are commonly costly, toxic, and unstable, cheaper, less toxic, and stable alternatives are highly sought after.

Three-dimensional (3D) printing offers the advantages of rapid prototyping and accurate fabrication of personalized support structures with the desired shape, thus, sodium alginate, chitosan, and halloysite nanotubes made via a 3D printer can form adsorption materials with different pore structures and porosity for desalination, water decontamination, and other applications [7]. The fused deposition modeling (FDM)-type 3D printer is one of the most popular 3D printers due to its affordability, low waste generation, potential for recycling, and user-friendliness. However, the potential of 3D-printed materials can be fully tapped only when they exhibit proper extrusion flow properties, are homogeneous, and can support their structure during and after printing. To be suitable for 3D printing, the composite-forming ink must have a sufficiently low viscosity for the extrusion process; however, this viscosity should be sufficiently high to limit ink dispersion during/after deposition to form a stable composite. These non-Newtonian fluids and shear-thinning material requirements can be met using cross-linkable inks. Sodium alginate (SA) features high biocompatibility, biodegradability, considerable cross-linking potential, high carboxyl and hydroxyl group content, and good thermal stability, and thus has numerous applications in the development of inks for 3D printing and the removal of dyes from wastewater [8]. Chitosan (CS) contains abundant amine and hydroxyl groups that can bind heavy metal ions and form electrostatic complexes with H+ under acidic conditions, and thus holds great promise as a natural adsorbent for the removal of heavy metals and dyes from wastewater [9]. Halloysite nanotubes (HNTs), which are mined from natural deposits and feature an aluminosilicate clay structure, are also regarded as promising adsorbents and are a cheaper and widely available alternative to carbon nanotubes [10]. Moreover, the rod-like geometry of HNTs prevents their entanglement and makes them (unlike carbon nanotubes) well dispersible in solutions and polymer matrices. Both SA and CS are abundantly available and cheap natural polymers that can be physically cross-linked to form amide bonds and thus enhance adsorption performance [11]. HNTs feature charged multilayer walls, i.e., a negatively charged outer surface bearing Si–OH groups and a positively charged inner surface bearing Al–OH groups [12].

It Is well known that the larger the specific surface area of the adsorbed substance, the stronger the adsorption force [13]. In this study, sodium alginate, chitosan, and halloysite nanotubes were combined with 3D-printed composite materials to prepare composite materials with different shapes and specific surface areas, which is expected to greatly improve their adsorption performance. Then, they were used as adsorbents to remove organic dyes from water and characterized by an instrumental technique. Taking the cationic dye MB as the model pollutant, the effects of the initial concentration of MB, pH, dosage of adsorbent, and contact time on adsorption properties were investigated. Finally, the adsorption isotherm and adsorption kinetics were fitted with different adsorption models to elucidate the adsorption mechanism. To date, the application of 3D printing in the manufacture of methylene blue (MB)-removal adsorbents in wastewater has not been reported.

## 2. Results

### 2.1. The Morphology of Composited Adsorbents

**Figure 1:**
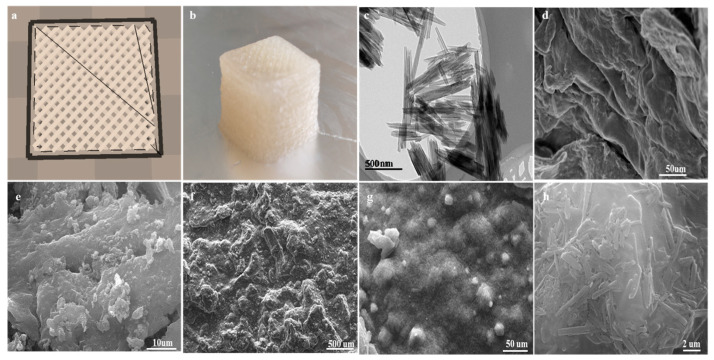
(**a**) The scheme of the printed cube (15 × 15 × 15 mm^3^) defined in CURA 15.04.6; (**b**) picture of real products; (**c**) TEM image of HNTs, (**d**–**f**) SEM images of SA, SA+CS, and SA+CS/HNTs respectively; (**g**) SA+CS/HNTs at low magnification; (**h**) SA+CS/HNTs at high magnification.

In the MES buffer solution, the activated carboxyl groups of SA reacted with the amino groups of CS to afford amide linkages. The amount of HNTs in the cross-linked polymer network increased with the reaction progress. In the SA–CS polymer network, HNTs and their functional groups experienced strong physical interactions. For example, MB molecules could engage in very strong electrostatic interactions with the composites, while the hydroxyl groups of SA could form Si–O bonds with Si in HNTs to stabilize the composite structure. Among the various techniques of 3D printing, the most common one is air pressure extrusion. In this case, the material is extruded during nozzle movement in three dimensions to result in layer-by-layer printing [14]. Figure 1a presents a cubic (15 × 15 × 15 mm^3^) model built using CURA 15.04.6 software. The optimal filling density of 50% was determined by performing a series of preliminary experiments. Overly small filling densities resulted in large gaps in the interior of the printed composite and small specific surface areas, which decreased adsorption efficiency. However, overly large filling densities resulted in small (or even zero) gaps in the composite interior (as the material itself has a certain width during the extrusion process), thereby leading to a small area of contact with the MB solution. At a filling density of 50%, the lines of yellow and the lines inside the composite intersected with each other to leave a certain gap, which greatly increased the area of contact with the MB solution and thus facilitated adsorption. In 3D printing, ink extrudability and printed structure stability are determined by ink rheology, while the printing process is affected by nozzle movement speed, extrusion rate, nozzle diameter/height, and layer [15]. Herein, preliminary experiments were conducted to ensure that the inks were extrudable. Figure 1b presents a representative image of a 3D-printed composite.

Figure 1c displays a TEM image of HNTs, revealing that these hollow nanotubes had a smooth surface and open ends. In view of their large surface area and abundant –OH and Si–O groups on the outer wall, HNTs can well adsorb various substances, offering the additional benefits of adequate dispersibility, high adsorption performance, high aspect ratio, sufficient biocompatibility, and low cost [16]. Figure 1d demonstrates that SA had a relatively smooth surface and a sheet-like structure. Figure 1e shows that upon the hybridization of SA with CS, the surface structure of the former was destroyed, and uneven porous particles were formed on the surface, indicating that SA and CS underwent amide coupling. In terms of adsorption performance, the convex surface structure was superior to the smooth surface structure. According to Figure 1f, the introduction of HNTs into the SA+CS complex resulted in surface coarsening. Figure 1g,h show that HNTs were present as nano-sized tubes and were uniformly dispersed. This uniform dispersion resulted in a high specific surface area and increased the permeability of the SA+CS polymer network to water [17,18]. The large surface area and abundant diffusion pathways of 3D-printed composites facilitated the aggregation of MB molecules at active sites to enhance adsorption efficiency.

### 2.2. FTIR Analysis

**Figure 2:**
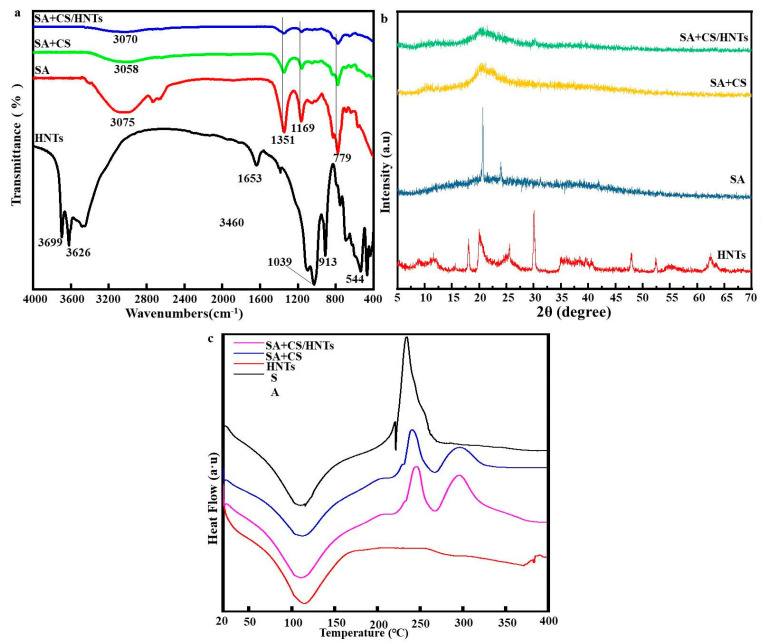
(**a**) FTIR of HNTs, SA, SA+CS, and SA+CS/HNTs; (**b**) XRD of HNTs, SA, SA+CS, and SA+CS/HNTs; (**c**) DSC of HNTs, SA, SA+CS, and SA+CS/HNTs.

Figure 2a presents the FTIR spectra of HNT, SA, SA+CS, and SA+CS/HNT composite inks. For HNTs, the bands at 3699 and 3626 cm^−1^ were ascribed to the stretches of Al_2_-OH units in which one OH group is connected to two Al atoms [19]. The bands at 3460 and 1653 cm^−1^ were ascribed to the O-H stretching and deformation vibrations of adsorbed water [20], respectively, while bands at 1039 and 913 cm^−1^ were ascribed to Si-O and Si-O-Si vibrations, respectively. The band at 544 cm^−1^ was attributed to the bending of Al_2_OH units due to the stretching of Si-O bonds [21]. For pure SA, the band at 3075 cm^−1^ was due to the stretching vibration of O-H bonds, while bands at 2739 and 2671 cm^−1^ were ascribed to the stretching of C-H bonds. Bands at 1351 and 1169 cm^−1^ were due to asymmetric and symmetric C=O stretching vibrations of SA, respectively. The bands at 779 and 573 cm^−1^ were related to antisymmetric C-O-C stretching and the C-O stretching vibration of uronic acid residues [22], respectively. Upon the introduction of CS into the SA polymer network, some bands of SA and CS disappeared, and characteristic new bands emerged. For example, the band at 3058 cm^−1^ was ascribed to the overlapping O-H and N-H stretching vibrations [23], while bands at 1351 and 1169 cm^−1^ were ascribed to N-H (amide II) and C-N (amide III) bond bending [24], respectively. The weak band at 1048 cm^−1^ was due to the angular vibration of -(CH_2_)_n_-CH_3_ units. The band at 694 cm^−1^ was due to C-O-C stretching and was typical of polysaccharides. The spectra of SA+CS/HNTs were not much different from that of SA+CS, which indicated that HNTs did not form new chemical bonds with SA+CS.

### 2.3. XRD Analysis

The XRD patterns of HNT, SA, SA+CS, and SA+CS/HNT composites are shown in Figure 2b. The characteristic peaks of HNTs were observed at 18.08°, 20.06°, 25.54°, and 30.16°. The characteristic sharp peak due to reflection from the basal plane of the HNT crystal structure appeared at a diffraction angle of 11.54° and (as follows from Bragg’s diffraction law) corresponded to an interplanar spacing of ~8.83 Å, which indicated that HNTs contained no water. The dehydrated state of HNTs was confirmed by the presence of peaks at 20.06° and 25.54° [25]. In the case of SA, we observed two strong peaks at 20.54° and 23.96° due to reflections from the (001) and (100) planes, respectively. The pattern of SA+CS featured a weakened SA peak at 20.54° and a new peak at 10.32°. The above findings show that positively charged CS and negatively charged SA engaged in the formation of hydrogen bonds and electrostatic interactions. Thus, the crystal structure of CS was concluded to change upon the formation of amide bonds with SA [26,27]. The patterns of SA+CS/HNT composites were different from those expected for a superposition of HNT and SA+CS patterns, as revealed by the position change and disappearance of some peaks. In this case, hybridization was concluded to result in HNT layer expansion and the disruption of ordered nanolayer stacking [28].

### 2.4. DSC Analysis

Figure 2c presents the DSC curves of HNT, SA, SA+CS, and SA+CS/HNT samples, revealing that in view of their large –OH, –COO–, and –NH group contents, samples containing SA and CS contained adsorbed water that was released upon heating. This loss of water was reflected by the appearance of an endothermic peak at ~110 °C, the enthalpy and position of which were related to the amount of bonded water and network structure, respectively [29]. For pure SA, an exothermic peak started to appear at ~200 °C, reaching its maximum at ~233 °C, i.e., the thermal behavior of SA was indicative of polymer disintegration [30,31]. The DSC curve of SA+CS featured a similar exothermic peak and also (unlike the curve of pure SA) exhibited exothermic peaks around 300 °C that were probably due to the formation of amide bonds between SA and CS. HNTs exhibited excellent thermal stability, and the corresponding DSC curve featured slight endothermicity with increasing temperature. The curve of SA+CS/HNT featured a gentler exothermic peak at ~300 °C than that of SA+CS, which reflected the good thermal stability and thermal barrier properties of HNTs.

### 2.5. Rheological Behavior

**Figure 3:**
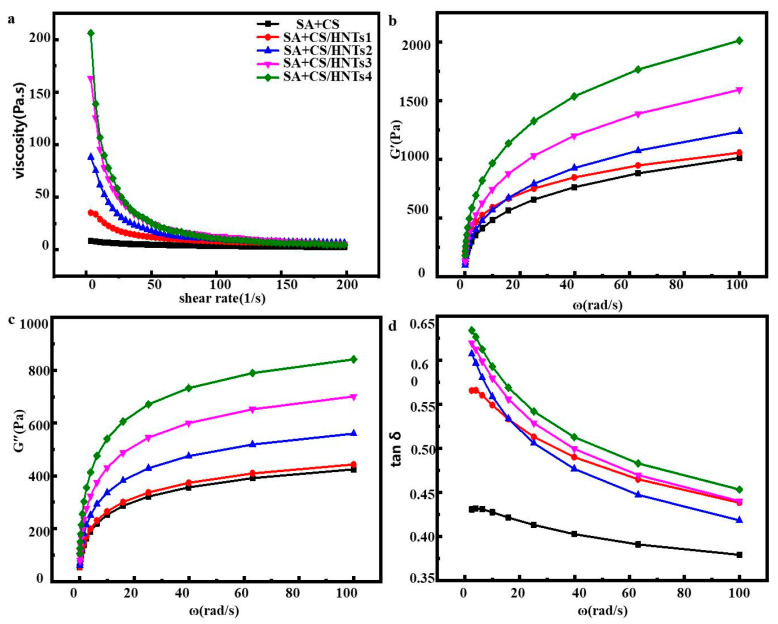
(**a**) Apparent viscosity, (**b**) G′, (**c**) G″, and (**d**) tanδ of the SA+CS mixture added with different HNT concentrations.

Ink viscosity is an important parameter determining ink extrudability [32]. Figure 3a shows the effect of shear rate on apparent ink viscosity at 25 °C, revealing that these parameters were negatively correlated. This behavior, which was ascribed to pseudoplastic fluid behavior and shear thinning, facilitated ink extrusion through the nozzle during printing. The dependence of ink viscosity on HNT content provides a handle allowing one to maintain the object shape during 3D printing. The elastic solid-like behavior is determined by the storage modulus (G′), which is a measure of elasticity, while the viscous response is expressed by the loss modulus (G″), which is the ratio of stress to strain used in dynamic oscillation frequency analysis. The prevalence of solid- or liquid-like responses is expressed by the loss tangent (tanδ = G″/G′) [33]. Solid-like elasticity is dominant at tanδ < 1 [34], while fluid-like properties are dominant at tanδ > 1. For all SA+CS/HNT inks, G′ exceeded G″ (Figure 3b,c), which was indicative of predominantly elastic behavior. In addition, both G′ and G″ increased with increasing HNT content at all oscillation frequencies. Figure 3d shows that all composites had tanδ < 1, i.e., the corresponding inks featured poor fluidity and exhibited solid-like behavior. Thus, stronger network structures were formed in inks with increasing HNT content.

### 2.6. Adsorption Performance

#### 2.6.1. Effect of pH

**Figure 4:**
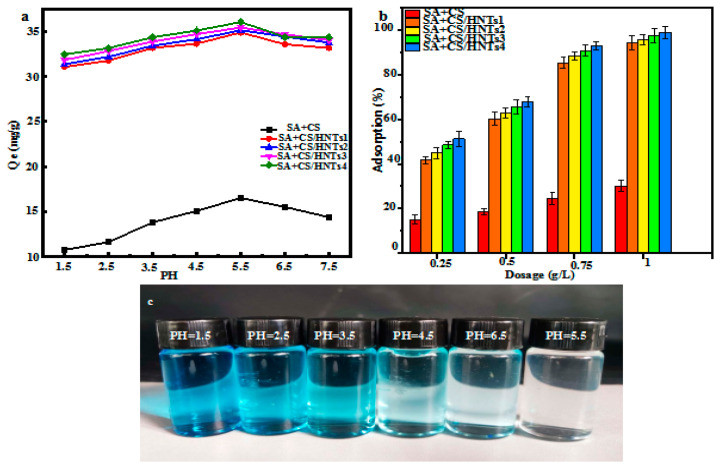
Influences of (**a**) pH and (**b**) 3D-printed composite adsorbent dosage on the adsorption of MB for SA+CS and SA+CS/HNTs; (**c**) the adsorption capacity of the SA+CS/HNTs4s composite in different pH solutions.

The pH value may affect the ionization degree of adsorbent functional groups as well as adsorbent surface charge and protonation degree [35]. At an initial MB concentration of 50 mg L^−1^, the MB adsorption capacity significantly increased as pH changed from 1.5 to 5.5, decreasing as pH was further increased to 7.5 (Figure 4a). Specifically, a change in pH from 1.5 to 5.5 resulted in an adsorption capacity increase from 10.8 to 15.6 mg g^−1^ (SA+CS), 31.1 to 34.9 mg g^−1^ (SA+CS/HNT1), 31.4 to 35.1 mg g^−1^ (SA+CS/HNT2), 31.8 to 35.4 mg g^−1^ (SA+CS/HNT3), and from 32.4 to 36.0 mg g^−1^ (SA+CS/HNT4), respectively (Figure 4b). Taking SA+CS/HNTs4 as an example, the above pH increase triggered an increase in MB adsorption capacity, and the solution, therefore, became less intensely colored and eventually turned colorless [36]. At pH > 5.5, the MB adsorption capacity decreased with increasing pH, and the solution color concomitantly became more intense (Figure 4c). At low pH, the competition between H^+^ and MB for adsorption sites became stronger, and the partial protonation of –NH_2_, –OH, and –COOH groups decreased adsorption capacity by hindering the formation of composite–MB complexes [35]. The slow decrease in MB removal efficiency with increasing pH may be due to the reduced competition between H^+^ and MB and the decreased protonation of composite functional groups. Therefore, pH 5.5 was chosen as optimal for further adsorption experiments because of the sufficient degree of functional group protonation and the reduced competition of MB with H^+^ for active sites under these conditions.

#### 2.6.2. Effect of Adsorbent Loading

The effects of adsorbent loading (0.25–1.00 g L^−1^) were probed at an initial MB concentration of 50 mg L^−1^, a contact time of 120 min, and a pH of 5.5. As the adsorbent loading increased from 0.25 to 1.00 g L^−1^, the removal efficiency increased from 15.1% to 30.0% (SA+CS), 41.7% to 94.2% (SA+CS/HNT1), 44.9% to 95.7% (SA+CS/HNT2), 48.6% to 97.5% (SA+CS/HNT3), and from 51.2% to 98.9% (SA+CS/HNT4) (Figure 4b). This behavior resembled that previously reported for similar dyes and was ascribed to the increase in the number of binding sites and surface area with increasing adsorbent loading [37].

#### 2.6.3. Effect of Initial MB Concentration

**Figure 5:**
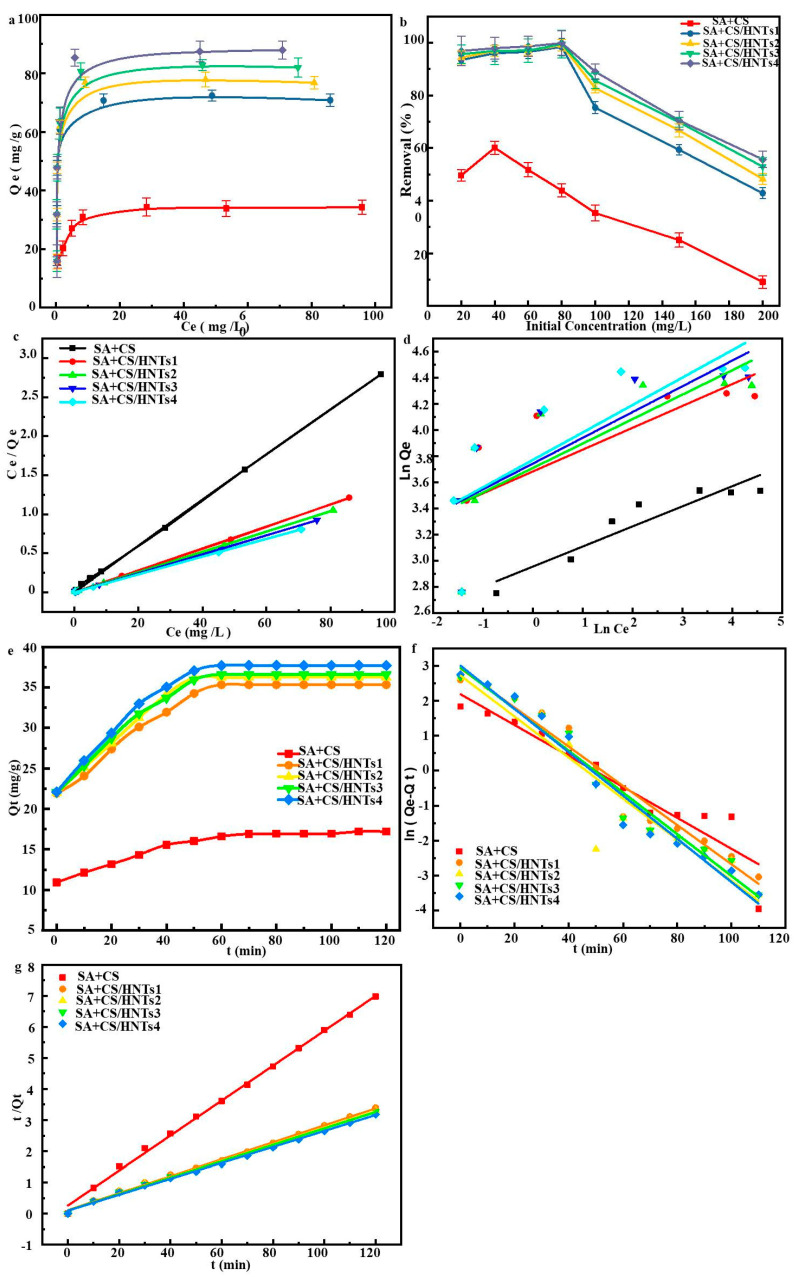
(**a**) Adsorption isotherm and (**b**) the effects of initial concentration on the removal rate of MB for the SA+CS and SA+CS/HNTs composites; (**c**) graph showing the adsorption isotherm model Langmuir isotherms plot; (**d**) Freundlich isotherms plot of SA+CS and SA+CS/HNTs composites; (**e**) adsorption kinetics and the corresponding (**f**) pseudo-first-order kinetic plots and (**g**) pseudo-second-order kinetic plots.

Figure 5a,b present the effects of initial MB concentration (C_0_) on adsorption isotherms and the removal rate, revealing that the adsorption capacity increased with increasing C_0_ and subsequently saturated, whereas an opposite trend was observed for the removal rate. At C_0_ = 80 mg L^−1^, Q_e_ reached a balanced state. Figure 5a shows that Q_e_ increased with increasing HNT content because of the increase in concomitants in the binding sites. At a low C_0_, the number of available binding sites is sufficient to adsorb MB from the aqueous solution, and the initial increase in MB removal efficiency with increasing C_0_ can be ascribed to the concomitant increase in the rate of MB mass transfer [38,39]. However, at a higher C_0_, the binding sites become saturated, and the adsorption driving force decreases. Figure 5b shows that maximal removal efficiencies (43.9%, 98.4%, 99.1%, 99.5%, and 99.8% for SA+CS, SA+CS/HNT1, SA+CS/HNT2, SA+CS/HNT3, and SA+CS/HNT4, respectively) were observed at C_0_ = 80 mg L^−1^.

### 2.7. Adsorption Isotherms

Adsorption isotherms can shed light on the adsorption mechanism (e.g., physisorption, chemisorption, and ion exchange) and allow one to determine the maximum adsorption capacity [40]. Experimental isotherms are usually fitted using several models, such as Langmuir and Freundlich. The former model assumes monolayer adsorption and considers the adsorption sites to be evenly distributed on the adsorbent surface [41], while the latter model assumes a single solute system and is often used in scenarios with high adsorbate concentrations.

Langmuir: C_e_/Q_e_ = C_e_/Q_m_ + (Q_m_K_L_)^−1^ (1)

Freundlich: lnQ_e_ = lnK_F_ + n^−1^lnC_e_ (2)

where Q_m_ (mg g^−1^) is the maximum adsorption capacity at equilibrium, n is related to adsorption intensity in the Freundlich model, K_L_ is a constant in the Langmuir model, and K_F_ is a constant in the Freundlich model.

Figure 5c,d present the results of adsorption isotherm fitting by Langmuir and Freundlich models, respectively, with the obtained parameters (e.g., R^2^) listed in Table 1. The adsorption of MB was better fitted by the Langmuir model than by the Freundlich model (R^2^ = 0.99926 vs. 0.85891, 0.99967 vs. 0.49118, 0.99968 vs. 0.52719, 0.99965 vs. 0.55996, and 0.99961 vs. 0.58077 for SA+CS, SA+CS/HNT1, SA+CS/HNT2, SA+CS/HNT3, and SA+CS/HNT4, respectively), i.e., MB adsorption occurred at specific uniformly distributed sites to form a monolayer [42]. This result, together with the involved electrostatic and hydrogen-bonding interactions, suggested that hydroxyl and carboxyl groups on the composite surface are occupied by MB up to saturation [43]. The Q_m_ value determined by Langmuir fitting is usually taken as an adsorption performance index, with higher values corresponding to better performance. Table 1 compares the MB adsorption performances of SA+CS/HNT composites with those of previously reported adsorbents, revealing that our composites outperformed most of their analogs. This high performance was attributed to the fact that the adsorption of MB on SA+CS/HNTs occurred via through-pore diffusion and involved active sites on the pore surface.

### 2.8. Adsorption Kinetics

Adsorption kinetics (which determine the time required to reach adsorption equilibrium) were fitted by quasi-first-order and quasi-second-order models. The former model is described as follows:

ln(Q_e_ − Q_t_) = lnQ_e_ − k_1_t (3)

where Q_t_ (mg g^−1^) is the adsorption capacity at time t, k_1_ (min^−1^) is the first-order rate constant, and t (min) is the contact time.

The latter model is described as follows:

t/Q_t_ = (k_2_Q_e_^2^)^−1^ + t/Q_e_ (4)

where k_2_ (g mg^−1^ min^−1^) is the second-order rate constant.

The evolution of adsorption capacity with time is presented in Figure 5e. For SA+CS/HNTs, the adsorption capacity rapidly increased during the first 40 min and then saturated at ~60 min. The high adsorption capacity of SA+CS/HNT composites was ascribed to their 3D network structure and abundant active sites (due to the presence of HNTs). Figure 5f,g show the results of fitting by quasi-first-order and quasi-second-order models, respectively, while Table 2 lists the corresponding parameters. The latter model (R^2^ = 0.9977, 0.9974, 0.9977, 0.9979, and 0.9979 for SA+CS, SA+CS/HNT1, SA+CS/HNT2, SA+CS/HNT3, and SA+CS/HNT4, respectively) performed better than the former model (R^2^ = 0.8920, 0.9595, 0.8809, 0.9637, and 0.9647 for SA+CS, SA+CS/HNT1, SA+CS/HNT2, SA+CS/HNT3, and SA+CS/HNT4, respectively). In addition, the Q_e_ values determined in the case of the quasi-first-order model (17.21, 35.59, 36.51, 36.82, and 37.90 mg g^−1^ for the SA+CS, SA+CS/HNT1, SA+CS/HNT2, SA+CS/HNT3, and SA+CS/HNT4, respectively) were approximately equal to those calculated for the quasi-second-order model (17.80, 36.79, 37.59, 37.99, and 39.12 mg g^−1^ for SA+CS, SA+CS/HNT1, SA+CS/HNT2, SA+CS/HNT3, and SA+CS/HNT4, respectively). The differences in adsorption capacity are closely related to the electrostatic interactions and the surface structure (e.g., surface area and pore volume) of the adsorbent [44]. Thus, the adsorption of MB on SA+CS/HNTs followed quasi-second-order kinetics and was driven by surface chemisorption.

### 2.9. Adsorption Mechanism

The possible adsorption mechanism presents mainly in the following two aspects: Firstly, 3D-printed sodium alginate/chitosan/halloysite nanotube composites have high porosity and interconnectivity between the pores, which provided more active sites, which are essential for good contact with MB, resulting in better adsorption capacity. Secondly, the carboxyl group (–COOH) of SA and the amino group (–NH_2_) of CS can react to form a stable amide bond (–NH–CO–), while the HNTs dispersed in the 3D network structure of SA+CS can form Si–O bonds with SA and CS by ultrasonic action. When in contact with MB^+^ ions, SA+CS/HNTs adsorbs them to the surface through strong electrostatic interactions until adsorption equilibrium is established.

### 2.10. Adsorbent Regeneration and Reusability

**Figure 6:**
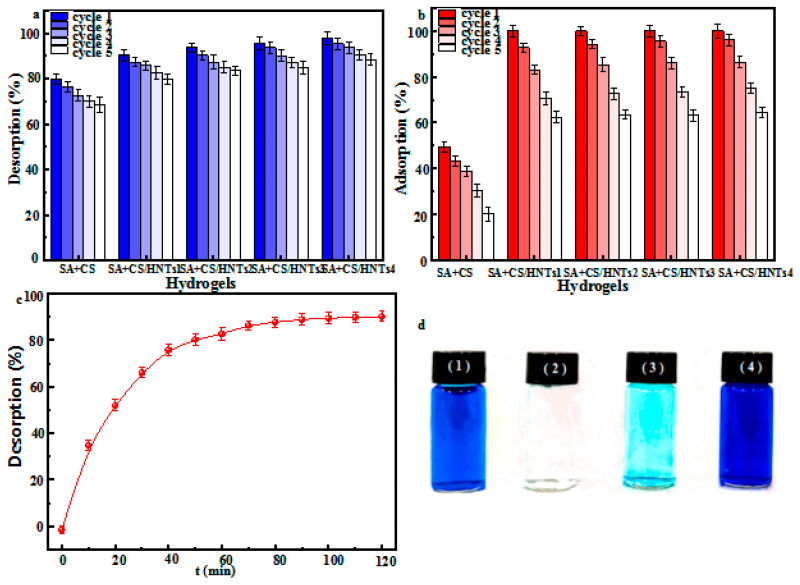
Subsequent desorption (**a**) and adsorption (**b**) of MB on the original SA+CS and SA+CS/HNTs composites. (**c**) Percentage of SA+CS/HNTs4 composite to MB desorption and (**d**) the adsorption–desorption of MB on the SA+CS/HNTs4 composite.

Adsorbent reusability and regeneration ability are important factors to consider in the case of large-scale usage. Herein, we tested the regeneration ability of SA+CS/HNTs by performing five consecutive adsorption–desorption cycles, revealing that desorption capacity decreased after each cycle (Figure 6a). Specifically, after the first adsorption–desorption cycle, the MB desorption efficiencies of SA+CS, SA+CS/HNT1, SA+CS/HNT2, SA+CS/HNT3, and SA+CS/HNT4 equaled 76.3%, 87.2%, 90.3%, 93.6%, and 95.3%, respectively, decreasing to 68.5%, 79.8%, 83.5%, 84.8%, and 88.5%, respectively, after five successive adsorption–desorption cycles. The relatively high value of 88.5% observed for SA+CS/HNT4 after five consecutive adsorption–desorption cycles was attributed to the strong interaction between MB and adsorbent functional groups. In addition, the adsorption efficiency also decreased after each adsorption–desorption cycle (Figure 6b), equaling 43.2%, 92.7%, 94.3%, 95.5%, and 96.1% for SA+CS, SA+CS/HNT1, SA+CS/HNT2, SA+CS/HNT3, and SA+CS/HNT4 after the first cycle, respectively, and decreasing to 20.3%, 62.4%, 63.6%, 63.1%, and 64.4%, respectively, after five cycles. MB adsorption–desorption capacity was not significantly changed after five cycles and was indicative of adequate regeneration ability. Ion exchange or electrostatic interactions likely led to the adsorption of MB at the composite surface, although hydrogen bonds also play an important role in the adsorption process [45]. Figure 6d(1–4) shows that upon exposure to SA+CS/HNT4, the blue MB solution (50 mg L^−1^) turned colorless after ~120 min. When the composite was exposed to 0.1 M HCl/ethanol, the solution became blue again after 60 min because of MB desorption. Figure 6c shows the evolution of MB desorption efficiency with time, demonstrating that ~80% of MB was desorbed in the first 60 min and that desorption efficiency slowly increased with increasing contact time. The above results suggest that the fabricated composites possess favorable desorption kinetics and reproducibility.

## 3. Materials and Methods

### 3.1. Materials

HNTs were obtained from Ningcheng Montmorillonite Co., Ltd. (Hohhot, Neimenggu, China). CS and SA were purchased from Shandong Aokang Biotech Co., Ltd. (Qingdao, Shandong, China). The substances 2-morpholinoethanesulfonic acid (MES), N-hydroxysuccinimide (NHS), MB (trihydrate), NaOH, ethanol, and hydrochloric acid (HCl) were sourced from Sinopod Holding Chongqing Chemical Reagent Co., Ltd. (Chongqing, China). All reagents/solvents were of analytical grade.

### 3.2. Preparation of SA+CS/HNT Composites

**Figure 7:**
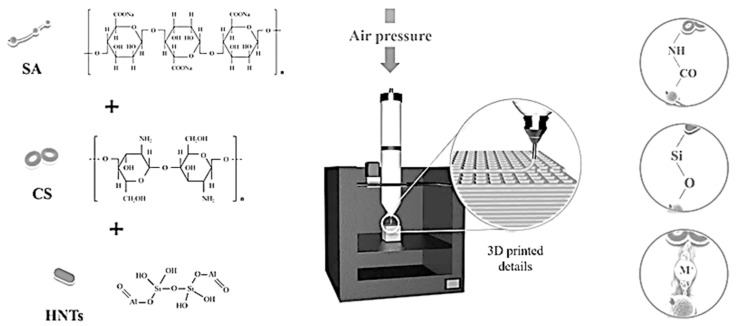
The preparation process of SA+CS/HNT composites.

SA (2 g) was dissolved in MES buffer to a concentration of 0.2 g L^−1^ through mechanical stirring for 3 h, and treated with EDC·HCl and NHS to activate carboxyl groups. Then, a 250 mL container with a mechanical agitator was charged with carboxylated CS and MES buffer (100 mL), and the reaction mixture was stirred at 450 rpm for 2 h. HNTs were dispersed in deionized water (50 mL) at 45 °C for ~15 min, and the dispersion was then added to the SA–CS system and stirred to afford an HNT loading of 0, 0.5, 1, 1.5, or 2 wt%. Finally, the obtained SA–CS/HNT ink (100 mL) was poured into a cylindrical barrel (internal diameter = 50 mm, height = 180 mm) and used for 3D printing, with extrusion performed using a preset program (Figure 7). The samples corresponding to the above loadings were denoted as SA+CS, SA+CS/HNT1, SA+CS/HNT2, SA+CS/HNT3, and SA+CS/HNT4, respectively.

### 3.3. Preparation of 3D-Printed Sodium Alginate/Chitosan/Halloysite Nanotube Composites

The 3D printer (model FlashForge, Creator Pro; Guangzhou, China) using FDM technology comprised three main components, namely the screw-pneumatic co-extrusion system, feeding system, and XYZ motion system. The movement and positioning of the ink during printing were controlled using a computer-customized JAVA program and microcontrollers with a minimized accuracy feature up to 0.01 mm. The printed parts had a cubic shape (15 × 15 × 15 mm^3^) and were designed using SolidWorks 2016. Printing was performed at 25 °C and controlled using CURA 15.04.6 (Ultimaker BV; Geldermalsen, The Netherlands) software. The print nozzle orifice (0.8 mm), layer height (0.4 mm), nozzle height (1 mm), packing density (50%), and pressure (4 kPa) had been optimized in preliminary experiments. The printed samples were stored at 4 °C in an Al box. Compared with ordinary single-screw extrusion or single pneumatic 3D-printing systems, our system supported the printing of higher-viscosity materials.

### 3.4. MB Adsorption onto 3D-Printed Composites

The effects of different factors were probed by changing one of the factors while leaving the others constant. Single-factor analysis was performed at pH = 5.5, sample volume = 40 mL, composite dose = 50 mg L^−1^, immersion time = 2 h, and MB concentration = 50 mg L^−1^. The obtained results were used to determine the best models for adsorption kinetics and isotherm fitting. Adsorption experiments were performed in a constant-temperature shaker (TS211-211B, Liang You, Changzhou, China) at an agitation rate of 250 rpm. The concentration of MB after adsorption was determined by UV spectrophotometry (752B, Jing Hua, Shanghai, China).

### 3.5. Transmission Electron Microscopy (TEM)

The sample (20 mg) was stirred in ethanol (3 mL) for 2 min, and the dispersion was ultrasonicated for 2 min. The suspension was drop-cast on a carbon-coated copper grid (400 mesh) through glass bottles and air-dried for 24 h. Electron micrographs were obtained using TEM (Philips CM200; Amsterdam, The Netherlands).

### 3.6. Scanning Electron Microscopy (SEM)

Sample surface morphology was probed by SEM (Hitachi, Japan) to determine the dispersion of HNTs in SA and the loading of CS.

### 3.7. Fourier Transform Infrared (FTIR) Spectroscopy

The functional groups of SA, CS, and HNTs, as well as the interactions between these components, were probed by FTIR spectroscopy (Suzhou Leiden Scientific Instrument Co., Ltd., Suzhou, China). Spectra were recorded at three random locations in the range of 400–4000 cm^−1^ using 32 scans and a resolution of 4 cm^−1^.

### 3.8. Rheological Measurements

Rheological measurements were performed using a rheometer (DHR 1TA, Waltham, MA, USA) with parallel plates (40 mm in diameter) separated by a gap of 1000 μm. The relationship between apparent viscosity and shear rate was determined at 25 °C in the range of 0.1–200 s^−1^; moreover, the steady flow properties of dispersions with different HNT contents were probed. Dynamic viscoelastic properties were investigated using a frequency oscillation range of 0.1–100 rad s^−1^ in the linear viscoelastic region. Each sample was measured in triplicate. The results were used to characterize the elastic modulus G′, loss modulus G″, and loss tangent tanδ = G″/G′ [46].

### 3.9. Differential Scanning Calorimetry (DSC)

Thermal properties were investigated by DSC (Perkin Elmer Pyris I, Waltham, MA, USA). In a typical DSC test, the sample (~10 mg, sealed in an Al pan) was heated from 20 to 400 °C at 10 °C min^−1^ under N_2_. An empty hermetic pan was used as a reference.

### 3.10. X-ray Diffraction (XRD) Analysis

XRD patterns (AXS D8 Advance, Bruker AXS GmbH, Karlsruhe, Germany) were recorded using Ni-filtered Cu Kα radiation at 30 mA and 40 kV in the 2θ range of 5–70° at a step size of 0.018° and a counting time of 1 s.

### 3.11. Adsorption Performance

For adsorption performance evaluations, each sample was placed into an MB solution (40 mL, 50 mg L^−1^), and the mixture was stirred at 250 rpm and at a room temperature of 25 °C. The absorbance of MB was measured with a UV spectrophotometer (752B, Jing Hua, Shanghai) over a period of 0–120 min and converted into MB concentration using a standard curve [47]. The equilibrium MB adsorption capacity Q_e_ (mg g^−1^) and MB removal efficiency R (%) were calculated as follows:

Q_e_ = (C_0_ − C_e_)V/m (5)

R = 100% × (C_0_ − C_e_)/C_0_ (6)

where C_0_ and C_e_ denote the initial and final (equilibrium) concentrations of MB, respectively, and V and m represent the volume of MB solution (L) and sample weight (g), respectively. Each adsorption experiment was performed in triplicate, and the experimental error was determined as ±2%.

We also probed the effect of MB concentration (20–200 mg L^−1^), pH (1.5–7.5, adjusted using 0.1 M HCl or NaOH), and adsorbent dosage (0.25 mg L^−1^) on adsorption performance. The pH end value of 7.5 was chosen because of the discoloration of MB solutions at higher pH levels.

To determine adsorption isotherms, we supplemented the MB solution (40 mL, 20–200 mg L^−1^) with the printed sample (50 mg) and stirred the dispersion until an adsorption equilibrium was established. The adsorption kinetics were probed under the same conditions, with sampling performed between 0 and 120 min.

### 3.12. Desorption and Recovery Experiments

After the completion of the adsorption experiment, the adsorbent was placed into a mixed HCl–ethanol solution (20 mL, 0.1 M) for 30 min and washed with deionized water to neutrality, thereby achieving a complete adsorption and desorption cycle. The regenerated composite was further used in the next cycle. Five adsorption–desorption cycles were performed, and the concentration of MB was spectrophotometrically measured in each cycle to determine the regeneration capacity (Rc, %) as follows:

Rc = 100% × (C_0_ − C_r_)/(C_0_ − C_e_) (7)

where C_r_ is the concentration of MB at desorption equilibrium (mg L^−1^).

### 3.13. Statistical Analysis

The final data were expressed as mean ± standard deviation. The significant differences are expressed as a value of *p* < 0.05. The SPSS 24.0 statistical analysis system was used for analysis of variance (ANOVA).

## 4. Conclusions

SA+CS/HNT composites were prepared by 3D printing and characterized. The high specific surface area and extensive microporous structure of these composites allow for effective removal of MB from wastewater. When the adsorbent load was 1 g L^−1^ and the concentration of MB was 80 mg L^−1^, the removal rate of MB was 80% and the maximum adsorption capacity was 376.3 mg g^−1^ after a 60 min treatment. The adsorption isotherms were best fitted by the Langmuir model, which was indicative of monolayer adsorption, while the adsorption kinetics were best fitted by a quasi-second order model, indicating the prevailing effect of external diffusion. After five adsorption–desorption cycles, the composite material maintains its adsorption properties to a large extent, and has great application prospects for the removal of MB in wastewater. Moreover, the SA+CS/HNT composites exhibited sufficient regeneration ability and were, therefore, concluded to hold great promise for MB removal from wastewater.

## Figures and Tables

**Table 1:** The fitting parameters of adsorption isotherms and kinetic models of different adsorbents.

Model	Parameters	Adsorbents				
		SA+CS	SA+CS/HNTs1	SA+CS/HNTs2	SA+CS/HNTs3	SA+CS/HNTs4
Langmuir model	R^2^	0.99926	0.99967	0.99968	0.99965	0.99961
	Q_m_ (mg/g)	89.7	267.4	321.7	357.3	376.3
	K_L_ (L/mg)	3.35	3.71	5.15	7.43	9.02
Freundlich model	R^2^	0.85891	0.49118	0.52719	0.55996	0.58077
	1/n	0.154	0.167	0.187	0.197	0.21
	K_F_ (L/mg)	19.19	39.77	40.85	42.26	43.46
Pseudo-first-order kinetics	R^2^	0.8920	0.95957	0.88096	0.96379	0.9647
	K_1_ (min^−1^)	0.04422	0.05603	0.05866	0.05963	0.06188
	Q_e_ (L/mg)	17.22	35.60	36.52	36.82	37.90
Pseudo-second-order kinetics	R^2^	0.99771	0.99749	0.99773	0.99791	0.99794
	K_2_ (g/mg/min)	1224.46	11,865.65	14,653.27	14,560.60	1615.30
	Q_e_ (L/mg)	17.8031	36.7918	37.5940	37.9939	39.1236

**Table 2:** The adsorption capacity of different adsorbents for MB.

Adsorbents	Maximum Adsorption Capacity for MB (mg/g)	Reference
SA+CS/HNTs composite	376.3	Our work
Betaine-modified Fe NPs	136	[38]
Fe_3_O_4_@SiO_2_-VTEOS-DMDAAC	109.9	[43]
CuO-Al_2_O_3_	97	[44]
Modified pine sawdust	84	[45]
Cotton fiber	113	[46]

## Data Availability

Data are contained within the article.
